# Neurometabolite Changes in Hyperthyroid Patients Before and After Antithyroid Treatment: An *in vivo* ^1^H MRS Study

**DOI:** 10.3389/fnhum.2021.739917

**Published:** 2021-11-26

**Authors:** Mukesh Kumar, Sadhana Singh, Poonam Rana, Pawan Kumar, Tarun Sekhri, Ratnesh Kanwar, Maria D’Souza, Subash Khushu

**Affiliations:** ^1^NMR Research Center, Institute of Nuclear Medicine and Allied Sciences (INMAS), New Delhi, India; ^2^Centre for Ayurveda Biology and Holistic Nutrition (CABHN), The University of Trans-Disciplinary Health Sciences and Technology, Bengaluru, India; ^3^Thyroid Research Center, Institute of Nuclear Medicine and Allied Sciences (INMAS), New Delhi, India

**Keywords:** hyperthyroidism, magnetic resonance spectroscopy, neurometabolites, dorsolateral prefrontal cortex, posterior parietal cortex

## Abstract

**Purpose:** Patients with hyperthyroidism have frequent neuropsychiatric symptoms such as lack of attention, concentration, poor memory, impaired executive functions, depression, and anxiety. These neurocognitive impairments such as memory, attention, and executive functions appear to be associated with dysfunction in brain regions. This study was conducted to investigate the metabolic changes in the brain subcortical regions, i.e., posterior parietal cortex and dorsolateral prefrontal cortex (DLPFC), in patients with hyperthyroidism before and after antithyroid treatment using proton magnetic resonance spectroscopy (^1^H MRS).

**Materials and Methods:** We collected neuropsychological and ^1^H MRS data from posterior parietal cortex and DLPFC, in both control (*N* = 30) and hyperthyroid (*N* = 30) patients. In addition, follow-up data were available for 19 patients treated with carbimazole for 30 weeks. The relative ratios of the neurometabolites were calculated using the Linear Combination Model (LCModel). Analysis of co-variance using Bonferroni correction was performed between healthy controls and hyperthyroid patients, and a paired *t*-test was applied in patients at baseline and follow-up. Spearman’s rank-order correlation was used to analyze bivariate associations between thyroid hormone levels and metabolite ratios, and the partial correlation analysis was performed between neuropsychological scores and metabolite ratios, with age and sex as covariates, in the patients before and after treatment.

**Results:** Our results revealed a significant decrease in choline/creatine [glycerophosphocholine (GPC) + phosphocholine (PCh)/creatine (tCr)] in both the posterior parietal cortex and DLPFC in hyperthyroid patients, and these changes were reversible after antithyroid treatment. The posterior parietal cortex also showed significantly reduced glutamate/creatine (Glu/tCr), (glutamate + glutamine)/creatine (Glx/tCr), and increased glutathione/creatine (GSH/tCr) ratios in the hyperthyroid patients over control subjects. In DLPFC, only (*N*-acetyl aspartate + *N*-acetyl aspartyl-glutamate)/creatine (NAA + NAAG)/tCr was increased in the hyperthyroid patients. After antithyroid treatment, (GPC + PCh)/tCr increased, and Glx/tCr decreased in both brain regions in the patients at follow-up. Gln/tCr in the posterior parietal cortex was decreased in patients at follow-up. Interestingly, (GPC + PCh)/tCr in DLPFC showed a significantly inverse correlation with free tri-iodothyronine (fT3) in hyperthyroid patients at baseline, whereas NAA/tCr showed positive correlations with fT3 and free thyroxine (fT4) in hyperthyroid patients before and after antithyroid treatment, in the posterior parietal cortex. In DLPFC, only (NAA + NAAG)/tCr showed positive correlations with fT3 and fT4 in the patients before treatment.

**Conclusion:** The overall findings suggest that all the brain metabolite changes were not completely reversed in the hyperthyroid patients after antithyroid treatment, even after achieving euthyroidism.

## Introduction

Thyroid hormones play an important role in the development and maturation of the brain and neuronal differentiation in humans (Zoeller and Rovet, 2004; Bernal, 2007). Dysfunction of these hormones may influence deficits in physiology, cognitive function, and emotional behavior (Bauer et al., 2008). Several studies have demonstrated that hyperthyroidism is commonly coupled with a range of neuropsychiatric symptoms, namely, nervousness, irritability, depression, anxiety, memory impairment, lack of concentration, and declined executive functions (Fahrenfort et al., 2000; Vogel et al., 2007; Yuan et al., 2019). The neurocognitive functions such as attention and concentration are associated with the cerebral cortex such as prefrontal and parietal cortices, which are highly sensitive to thyroid hormone concentration (Heuer, 2007). Although the effect of adult-onset hyperthyroidism on these brain regions is known, the underlying mechanism for the brain dysfunction and its reversibility remains unclear. We speculate that excess thyroid hormone not only influences brain function but may lead to metabolic abnormalities in the mature brain structures, namely, posterior parietal cortex and dorsolateral prefrontal cortex (DLPFC), critical for cognitive functions.

Magnetic-resonance-based imaging and spectroscopy have enormous potential for understanding structural, functional, and metabolic changes in various brain regions. Among non-invasive techniques, proton magnetic resonance spectroscopy (^1^H MRS) provides an *in vivo* quantification of various neurochemicals/metabolites during pathological conditions in humans and animal models. Several studies have reported metabolic changes in different brain regions in hyperthyroid patients using ^1^H MRS (Bhatara et al., 1998; Elberling et al., 2003; Danielsen et al., 2008). A previous ^1^H MRS study demonstrated a decreased choline/creatine (Cho/Cr) ratio in the right prefrontal cortex in patients with hyperthyroidism (Bhatara et al., 1998). Elberling et al. (2003) have shown significantly reduced total choline and myoinositol (mI) in the acute phase of Graves’ thyrotoxicosis compared with the healthy volunteers. Similarly, Danielsen et al. (2008) have reported significantly reduced *N*-acetyl aspartate (NAA), creatine (Cr), Cho, mI, and combined glutamate and glutamine (Glx) in both white matter (WM) and gray matter (GM) in parieto-occipital regions in patients with Graves’ disease, and also found a reversal of Cho/Cr ratio in the patients at follow-up after the acute phase of the disease. However, there are limited studies that reveal the metabolic changes associated with hyperthyroidism in the posterior parietal cortex and DLPFC of the brain.

In this study, we aimed to examine the metabolic changes in the brain subcortical regions, i.e., posterior parietal cortex and DLPFC, in patients with hyperthyroidism before and after antithyroid treatment using ^1^H MRS. Furthermore, the correlations of clinical indices, neuropsychological scores, and MRS data in the hyperthyroid patients were also carried out to understand the impact of clinical scores in these patients.

## Materials and Methods

### Study Design

The hyperthyroid patients underwent thyroid hormone tests, neuropsychological assessments, and ^1^H MRS scanning pre and post 30 weeks of carbimazole treatment (after achieving a euthyroid state). The dose of carbimazole for each patient was decided by the clinician according to the disease severity during the period of antithyroid treatment. A starting dose of carbimazole 0.5 mg/kg/day was given and patients were monitored every 6 weeks for free tri-iodothyronine (fT3), free thyroxine (fT4), and thyroid-stimulating hormone (TSH) levels. The dose was titrated to keep fT4 in the lower half of the normal range (12–22 pmol/l). After 30 weeks of antithyroid treatment, patients were ordered back to undergo comprehensive assessments to evaluate the therapeutic efficacy. The healthy control group underwent the same assessments one time only, at the beginning of the study.

### Subjects

We recruited 30 healthy controls and 30 hyperthyroid patients for this study. Out of the original cohort of 30 patients, 19 were included in the follow-up study. Demographic, clinical, and neuropsychological data for all the subjects are summarized in Table 1. All the hyperthyroid patients were diagnosed with hyperthyroidism for the first time and were recruited from the Thyroid Research Centre of our Institute. All control subjects chosen for the study were recruited from the local community. Thyroid function tests [TSH, fT3, and fT4] were carried out in all the patients and control subjects.

**TABLE 1:** Demographic, clinical and neuropsychological characteristics of all the subjects.

Characteristics	Controls (*N* = 30)	Hyperthyroid (*N* = 30)	Hyperthyroid (*n* = 19)		*p-* value	*p-* value
			Pre-therapy	Post-therapy		
Gender (Male/Female)	9/21	8/22			0.197	
Age (Years)	31.70 ± 8.24	34.28 ± 7.12			0.12	
Education (Years)	13.17 ± 3.30	12.37 ± 3.90			0.396	
BMI (Kg/m^2^)	22.60 ± 3.39	22.51 ± 3.95	21.29 ± 2.79	22.58 ± 2.45	0.925	
TSH (μiU/ml)	2.41 ± 0.73	0.014 ± 0.017	0.010 ± 0.006	1.93 ± 1.75	0.000^a^	0.004^b^
fT4 (pmol/liter)	15.78 ± 4.93	44.28 ± 19.92	46.95 ± 19.88	16.44 ± 4.70	0.00^a^	0.000^b^
fT3 (pmol/liter)	5.83 ± 2.01	15.83 ± 6.63	17.56 ± 6.80	4.60 ± 1.98	0.000^a^	0.000^b^
**Memory scale**						
Long term episodic memory	5 ± 0	4.72 ± 0.65	4.74 ± 0.73	4.89 ± 0.32	0.10	0.47
Recent episodic memory	5 ± 0	4.93 ± 0.26	5.00 ± 0.00	4.95 ± 0.23	0.042^a^	0.12
Mental balance	4.48 ± 0.69	3.86 ± 1.33	3.84 ± 1.34	4.05 ± 1.31	0.05^a^	0.61
Working memory span (forward and backward)	4.72 ± 0.45	4.17 ± 1.20	4.16 ± 1.34	4.84 ± 0.69	0.029^a^	0.06
Delayed Recall	4.76 ± 0.44	4.55 ± 0.95	4.53 ± 0.96	4.68 ± 0.75	0.30	0.37
Immediate Recall	4.83 ± 0.38	4.62 ± 0.82	4.63 ± 0.76	4.84 ± 0.37	0.13	0.36
Immediate recall of semantically related word pairs	4.93 ± 0.26	4.59 ± 0.82	4.68 ± 0.75	4.95 ± 0.23	0.097	0.22
Immediate recall of arbitrarily related word pairs	4.69 ± 0.66	4.38 ± 1.05	4.00 ± 1.29	4.95 ± 0.23	0.16	0.02^b^
Visual retention	4.76 ± 0.44	4.21 ± 1.21	4.00 ± 1.29	4.95 ± 0.23	0.036^a^	0.009^b^
Recognition of objects	4.90 ± 0.31	4.17 ± 1.10	4.16 ± 1.21	4.74 ± 0.45	0.017^a^	0.04^b^
**Performance Tests of intelligence**						
Performance quotient	115.86 ± 20.02	104.34 ± 21.12	100.78 ± 17.23	121.21 ± 17.49	0.038^a^	0.001^b^
P/K × 100	133.92 ± 85.67	151.51 ± 104.69	171.54 ± 122.02	149.78 ± 67.70	0.528	0.36
Nahor-Benson Test	0.66 ± 1.01	1.71 ± 2.05	1.95 ± 2.07	0.53 ± 0.90	0.02^a^	0.002^b^
Bender Visual-Motor Gestal Test	1.31 ± 1.97	7.75 ± 8.61	8.84 ± 9.22	4.53 ± 3.64	0.004^a^	0.048^b^
MMSE	28.59 ± 1.68	26.62 ± 2.87	26.58 ± 2.76	27.11 ± 2.16	0.002^a^	0.44

*Mean and standard deviation (SD) of demographic, thyroid hormone levels, neuropsychological tests scores.*

*^a^Independent sample t-test between hyperthyroid and controls (p < 0.05); ^b^Paired t-test between pre-therapy vs. post-therapy in 19 hyperthyroid patients (p < 0.05). BMI, body mass index; TSH, thyroid stimulating hormone; fT4, free thyroxine; fT3, free tri-iodothyronine; P/K, ratio of Pass-a-long and Koh’s test; MMSE, Mini Mental State Examination.*

The study was approved by the institutional research ethics committee, and informed consent was obtained for all the participants. Exclusion criteria for patients and controls were clinical evidence of stroke, head injury, cardiovascular diseases, history of smoking, alcohol or drug dependence, psychiatric disorders, or cognitive impairment.

### Neuropsychological Data

The Mini Mental State Examination (Folstein et al., 1975) and the “Postgraduate Institute Battery of Brain Dysfunction (PGIBBD)” (Pershad and Verma, 1990) neuropsychological tests were performed in all the subjects. The memory scale, a part of PGIBBD, consists of a series of domain-specific cognitive function tests such as long-term episodic memory, recent episodic memory, mental balance, working memory span, delayed and immediate recall, immediate recall of semantically related word pairs, immediate recall of arbitrarily related word pairs, visual retention, and recognition of the objects (Pershad and Verma, 1990), were conducted for each subject. The higher raw score of each memory function test indicated better performance.

For executive function, Koh’s block design (KBD) and pass-a-long test (PALT) tests were performed in both the controls and patients. The higher the raw scores of KBD and PALT, the better the performance of the individual. For visuospatial and motor functions, the Bender–Gestalt test (BGT) and Nahor–Benson test (NBT) were used. The higher raw dysfunction scores indicated poor performance.

The scoring of all neuropsychological data was done as per the procedure, which has been described in detail elsewhere (Pershad and Verma, 1990).

### *In vivo* MRI/MRS Acquisition

We used 3T whole-body magnetic resonance (MR) system (Magnetom Skyra, Siemens, Germany) with a 20-channel head coil and a 45 mT/m actively shielded gradient system for brain studies. High-resolution T1-weighted images were collected using a Magnetization Prepared Rapid Acquisition Gradient-Echo (MPRAGE) pulse sequence [repetition time (TR) = 1,900 ms; echo time (TE) = 2.49 ms; matrix size = 256 × 256; field of view (FOV) = 240 × 240 mm^2^; slice thickness = 0.9 mm; and number of slices = 160]. Anatomical imaging was performed in all three orthogonal planes for the positioning of the MRS voxels. We collected T2-weighted multislice images [TR = 5,600 ms, TE = 100 ms, number of excitations (NEX) = 2, matrix size = 312 × 512, FOV = 220 mm, 25 slices, slice thickness = 4.0 mm, and distance factor = 1.2 mm] covering the entire brain, to rule out the presence of any focal brain lesions.

**FIGURE 1:**
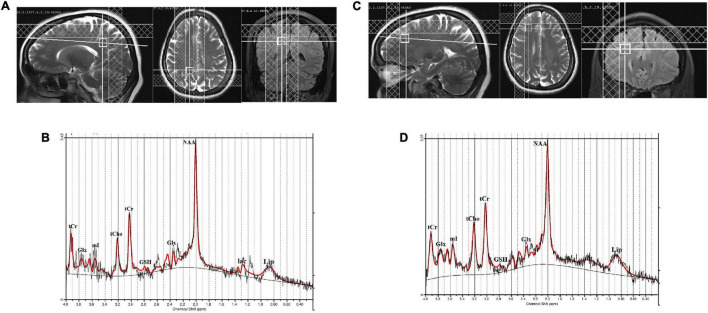
**(A)** Location of 12 × 12 × 12 mm^3^ voxel in the right posterior parietal cortex, **(B)** a representative spectrum analyzed by LCModel, **(C)** location of 12 × 12 × 12 mm^3^ voxel in the right dorsolateral prefrontal cortex (DLPFC), and **(D)** a representative spectrum analyzed by LCModel.

The MRS was acquired using the single volume point resolved spectroscopy sequence (PRESS) with acquisition parameters: TR/TE = 2,000/30 ms; spectral points = 1,024; spectral bandwidth = 1,200 Hz; and averages = 256). Water suppression was achieved with a chemically selective suppression (CHESS) pulse sequence. Automated global shimming was used to minimize the *B*_0_ inhomogeneities and localized shimming was done to further minimize *B*_0_ field variations over the voxel of interest. Water unsuppressed spectra with 10 averages were also acquired for reference purposes. The line width of spectra in all subjects was less than 0.156 ppm (20 Hz) at full-width at half maximum (FWHM). For the right posterior parietal cortex, a voxel of 12 × 12 × 12 mm^3^ was placed in a sagittal slice and visually inspected on axial and coronal MR images. The visual cortex at the caudal pole of the brain and the somatosensory cortex just behind the central sulcus were used as anatomical boundaries for the voxel placement (Figure 1A). Similarly, a voxel of 12 × 12 × 12 mm^3^ was placed in the right DLPFC, which was identified on the sagittal and coronal MR images (Figure 1C). For the right DLPFC, the superior frontal sulcus, the lateral fissure, and the genu of the corpus callosum were used as anatomical boundaries for the voxel placement. The position of the voxels was visually inspected and adjusted based on identifiable anatomical landmarks about standard brain atlases (Jackson and Duncan, 1996).

### Magnetic Resonance Spectroscopy Data Analysis

The MRS raw data were processed using LCModel Version 6.3 for quantitative assessment of the brain metabolites (Provencher, 1993, 2001). To determine the detectability and quantitative reliability of each metabolite from the measured spectra, the % SD of each metabolite, defined as the Cramer–Rao lower bound (CRLB) criteria, was derived as a measurable index for quantitative reliability (Provencher, 1993). CRLB ≤ 20% was considered as an acceptable level of quantitative reliability. Metabolite ratios were used to reduce the variability in absolute values for different metabolites as observed in the subjects. We used total creatine (tCr) values as the internal reference for relative quantification due to its relatively stable concentration in the brain (Provencher, 2001).

### Statistical Analysis

All the statistical analyses were conducted using SPSS (version 20.0, SPSS Inc., Chicago, IL, United States) statistical software. Demographic, clinical, and neuropsychological parameters were assessed by independent samples *t*-tests, and categorical characteristics were compared using the chi-squared test. Analysis of co-variance using Bonferroni correction was performed to compare different metabolite ratios between the two groups with age and sex as covariates. A paired *t*-test was used to compare metabolite ratios in the patients at baseline and the follow-up. MRS voxel GM, WM, and cerebrospinal fluid (CSF) were calculated using the Matlab script (Quadrelli et al., 2016).

Spearman’s rank-order correlation was used to analyze bivariate associations between clinical indices (fT3, fT4, and TSH) and metabolite ratios, and the partial correlation analysis was performed between neuropsychological scores and metabolite ratios, with age and sex as covariates, in the healthy controls and patients before and after treatment. A *p* value ≤ 0.05 was considered to be statistically significant.

## Results

### Demographic, Clinical, and Neuropsychological Variables

Demographic, clinical, and neuropsychological data of hyperthyroid patients and control subjects are summarized in Table 1. No significant differences in age (*p* = 0.120), sex (*p* = 0.197), education (*p* = 0.396), or BMI (*p* = 0.925) appeared between the groups. In hyperthyroid patients, suppressed TSH (≤0.01 μIU/ml), elevated fT3 (≥7 pmol/l) and fT4 (≥22 pmol/l) levels were observed. However, the thyroid hormones of the control subjects were within normal ranges (TSH = 0.27–4.2 μIU/ml, fT3 = 2.6–6.8 pmol/l, and fT4 = 12.0–22.0 pmol/l). After antithyroid treatment, a significant increase in TSH levels (1.93 ± 1.75 μIU/ml), and reduced fT3 (4.60 ± 1.98 pmol/l) and fT4 (16.44 ± 4.70 pmol/l) values, were found in the patients at follow-up.

Significantly decreased scores were observed in recent episodic memory, mental balance, working memory span, visual retention, recognition of objects, performance quotient, and increased scores in NBT and BGT, in hyperthyroid patients over healthy controls (Table 1). After antithyroid treatment, patients at follow-up showed improvement in working memory span, immediate recall of arbitrarily related word pairs, visual retention, recognition of objects, performance quotient, and NBT and BGT scores (Table 1).

### Magnetic Resonance Spectroscopy Results

The quality of the spectra between the groups was not significantly different as judged by FWHM and signal-to-noise ratio (SNR). The FWHM and SNR of the healthy controls and hyperthyroid patients before and after treatment are summarized in Table 2. No significant differences for GM, WM, and CSF in voxel tissue components were found between healthy controls, hyperthyroid patients before and after thyroxine treatment in both the brain regions (Table 2). There was no significant difference in tCr concentration between healthy controls and hyperthyroid patients in the right posterior parietal cortex (*p* = 0.96) and right DLPFC (*p* = 0.39). In the posterior parietal cortex, significantly decreased Glu/tCr (*p* = 0.001), [glycerophosphocholine (GPC) + phosphocholine (PCh)]/tCr (*p* = 0.004), and Glx/tCr (*p* = 0.011), and increased GSH/tCr (*p* = 0.035) metabolite ratios were found in hyperthyroid patients over healthy controls (Table 2). In addition, NAA/tCr (*p* = 0.075) showed an increasing trend in hyperthyroid patients compared to healthy controls, but it did not reach the level of statistical significance. In DLPFC, significantly reduced (GPC + PCh)/tCr (*p* = 0.005) and increased (NAA + NAAG)/tCr (*p* = 0.030) were observed in hyperthyroid patients compared to healthy controls (Table 2). The representative spectra acquired from the right posterior parietal cortex and right DLPFC regions of a control subject are shown in Figures 1B,D.

**TABLE 2:** Relative values of neuro-metabolites and spectral quality measures for the 30 controls, and 30 hyperthyroid and 19 hyperthyroid patients at baseline and at follow-up in posterior parietal cortex and dorsolateral prefrontal cortex (DLPFC).

(Metabolites) Posterior parietal cortex (PP)	Control (*n* = 30) (Mean ± SD)	Hyperthyroidism (*n* = 30) (Mean ± SD)	Hyperthyroidism (*n* = 19)		*p*-value	*p-*value	CRLB
			Pre-therapy (Mean ± SD)	Post-therapy (Mean ± SD)			
Gln/tCr	1.495 ± 0.33	1.354 ± 0.38	1.452 ± 0.399	1.107 ± 0.346	0.162	0.020^b^	≤20 %
Glu/tCr	1.156 ± 0.29	0.897 ± 0.21	0.940 ± 0.227	1.021 ± 0.177	0.001^a^	0.225	≤20 %
GSH/tCr	0.257 ± 0.05	0.287 ± 0.074	0.278 ± 0.071	0.333 ± 0.087	0.033^a^	0.058^b^	≤20 %
mI/tCr	1.439 ± 0.27	1.364 ± 0.32	1.334 ± 0.262	1.193 ± 0.467	0.303	0.149	≤11 %
NAA/tCr	1.431 ± 0.20	1.484 ± 0.20	1.480 ± 0.168	1.469 ± 0.202	0.077^a^	0.831	≤10 %
GPC+PCh/tCr	0.283 ± 0.04	0.249 ± 0.03	0.258 ± 0.043	0.302 ± 0.038	0.004^a^	0.006^b^	≤8 %
NAA+NAAG/tCr	1.652 ± 0.16	1.664 ± 0.17	1.700 ± 0.194	1.762 ± 0.195	0.441	0.111	≤5 %
Glu+Gln/tCr	2.585 ± 0.46	2.225 ± 0.45	2.356 ± 0.499	1.856 ± 0.477	0.011^a^	0.035^b^	≤12 %
**Dorsolateral prefrontal cortex (DLPFC)**							
Gln/tCr	1.259 ± 0.235	1.171 ± 0.355	1.377 ± 0.404	1.324 ± 0.340	0.174	0.610	≤20 %
Glu/tCr	0.930 ± 0.222	0.992 ± 0.251	0.985 ± 0.232	1.076 ± 0.198	0.124	0.155	≤20 %
GSH/tCr	0.312 ± 0.058	0.303 ± 0.082	0.300 ± 0.074	0.329 ± 0.086	0.580	0.323	≤20 %
mI/tCr	1.381 ± 0.260	1.311 ± 0.310	1.245 ± 0.249	1.211 ± 0.414	0.266	0.661	≤10 %
NAA/tCr	1.346 ± 0.220	1.305 ± 0.242	1.323 ± 0.213	1.329 ± 0.255	0.973	0.914	≤10 %
GPC+PCh/tCr	0.290 ± 0.047	0.249 ± 0.036	0.251 ± 0.033	0.309 ± 0.039	0.005^a^	0.001^b^	≤7 %
NAA+NAAG/tCr	1.519 ± 0.127	1.574 ± 0.176	1.553 ± 0.176	1.551 ± 0.187	0.028^a^	0.943	≤5 %
Glu+Gln/tCr	2.138 ± 0.302	2.118 ± 0.444	2.299 ± 0.447	2.006 ± 0.520	0.730	0.037^b^	≤14 %

**Spectral Quality Measures**							

**Posterior parietal cortex (PP)**							
FWHM (in ppm)	0.059 ± 0.02	0.062 ± 0.02	0.063 ± 0.025	0.057 ± 0.02	0.48	0.52	
SNR	12.13 ± 2.43	11.77 ± 1.19	12 ± 2.09	12.53 ± 2.48	0.52	0.36	
GM	0.376 ± 0.11	0.416 ± 0.14	0.438 ± 0.15	0.377 ± 0.13	0.21	0.20	
WM	0.598 ± 0.13	0.553 ± 0.17	0.531 ± 0.19	0.594 ± 0.15	0.21	0.25	
CSF	0.031 ± 0.05	0.031 ± 0.04	0.033 ± 0.04	0.030 ± 0.02	0.5	0.81	
**DLPFC**							
FWHM (in ppm)	0.064 ± 0.02	0.065 ± 0.026	0.059 ± 0.027	0.069 ± 0.02	0.96	0.183	
SNR	12.56 ± 1.95	12.41 ± 2.03	12.89 ± 2.07	11.79 ± 2.46	0.79	0.074	
GM	0.385 ± 0.12	0.416 ± 0.14	0.394 ± 0.16	0.371 ± 0.13	0.37	0.61	
WM	0.591 ± 0.13	0.553 ± 0.17	0.583 ± 0.20	0.595 ± 0.15	0.34	0.82	
CSF	0.024 ± 0.02	0.031 ± 0.04	0.024 ± 0.04	0.030 ± 0.02	0.42	0.55	

*^a^Indicates p < 0.05 (Bonferroni correction for multiple comparison between control and hyperthyroid patients); ^b^indicates p < 0.05 (paired t-test in hyperthyroid patients at baseline and at follow-up). SD, standard deviation; CRLB, Cramer-Rao lower bound; FWHM, full width at half maximum; SNR, signal to noise ratio; GM, gray matter; WM, white matter; CSF, cerebrospinal fluid.*

After treatment, (GPC + PCh)/tCr metabolite ratio reversal (*p* = 0.006 for right posterior parietal cortex; *p* = 0.001 for right DLPFC) in both the brain regions studies. Patients at follow-up showed significantly decreased Gln/tCr (*p* = 0.020), Glx/tCr (*p* = 0.035) in the posterior parietal cortex, and Glx/tCr (*p* = 0.037) in DLPFC. Also, GSH/tCr (*p* = 0.058) showed an increasing trend in the patients at follow-up (Table 2).

### Correlation Analysis Among Thyroid Indices, Neuropsychological Scores, and Neurometabolites

**FIGURE 2:**
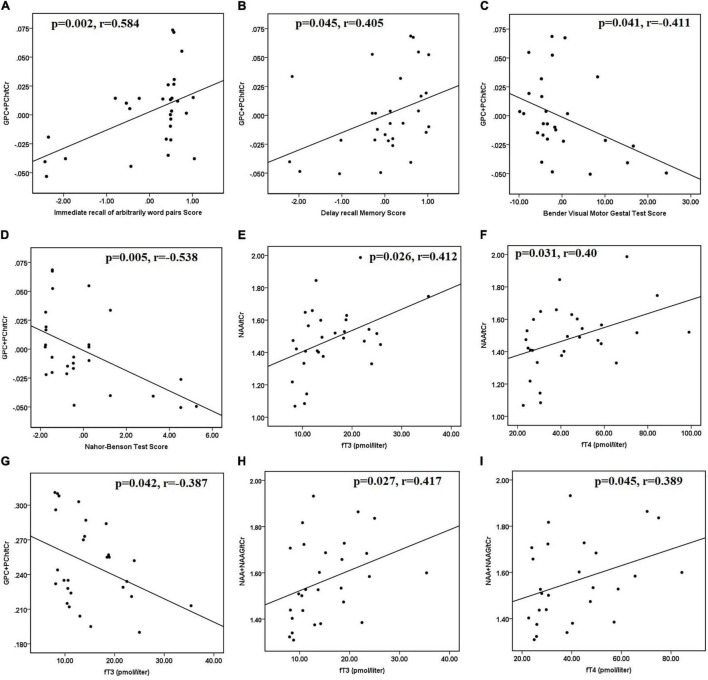
Scatter plots showing significant correlations between clinical data, neuropsychological scores, and metabolite ratios in hyperthyroid patients at baseline. **(A)** Positive correlation between (GPC + PCh)/tCr in posterior parietal cortex and immediate recall of arbitrarily related word pairs scores, **(B)** positive correlation between (GPC + PCh)/tCr in DLPFC and delayed recall memory scores, negative correlations between **(C)** (GPC + PCh)/tCr in DLPFC and Bender Visual-Motor Gestal test scores, **(D)** (GPC + PCh)/tCr in DLPFC and Nahor–Benson test scores, in hyperthyroid patients at baseline. Positive correlations between **(E)** NAA/tCr in posterior parietal cortex and fT3 and **(F)** NAA/tCr in posterior parietal cortex and fT4. **(G)** Negative correlation between (GPC + PCh)/tCr in DLPFC and fT3, positive correlations between **(H)** (NAA + NAAG)/tCr in DLPFC and fT3, and **(I)** (NAA + NAAG)/tCr in DLPFC and fT4, in hyperthyroid patients at baseline. DLPFC, right dorsolateral prefrontal cortex; GPC, glycerophosphocholine; NAA, *N*-acetyl aspartate; NAAG, *N*-acetyl aspartyl-glutamate; PCh, phosphocholine; tCr, total creatine.

After controlling for age and sex, hyperthyroid patients had positive correlations between (GPC + PCh)/tCr in the posterior parietal cortex and immediate recall of arbitrarily related word pairs (*r* = 0.584 and *p* = 0.002) (Figure 2A). In hyperthyroid patients, (GPC + PCh)/tCr in DLPFC positively correlated with delayed recall memory scores (*r* = 0.405 and *p* = 0.041) (Figure 2B), and negatively with Bender Visual-Motor Gestal test (*r* = −0.416 and *p* = 0.04) (Figure 2C) and Nahor–Benson test (*r* = −0.538 and *p* = 0.005) scores (Figure 2D).

**FIGURE 3:**
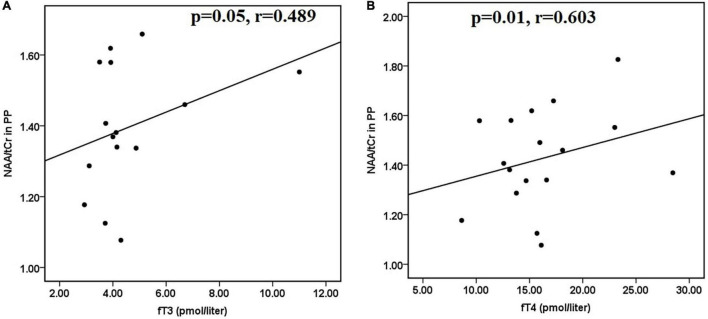
Scatter plots showing significant positive correlations between **(A)** NAA/tCr in posterior parietal cortex and fT3 and **(B)** NAA/tCr in posterior parietal cortex and fT4, in hyperthyroid patients at follow-up. NAA, *N*-acetyl aspartate; tCr, total creatine.

Furthermore, in hyperthyroid patients at baseline, NAA/tCr in the posterior parietal cortex showed a significantly positive correlation with fT3 (*r* = 0.412 and *p* = 0.026) (Figure 2E) and fT4 (*r* = 0.400 and *p* = 0.031) (Figure 2F). GPC + PCh/tCr in DLPFC showed an inverse correlation with fT3 (*r* = −0.387 and *p* = 0.042) (Figure 2G), and positive correlations between (NAA + NAAG)/tCr and fT3 (*r* = 0.417 and *p* = 0.027) (Figure 2H) and fT4 (*r* = 0.389 and *p* = 0.045) (Figure 2I) in hyperthyroid patients at baseline. At follow-up, only NAA/tCr showed a significantly positive correlation with fT3 (*r* = 0.489 and *p* = 0.05) (Figure 3A) and fT4 (*r* = 0.603 and *p* = 0.01) (Figure 3B) in the posterior parietal cortex in patients after antithyroid treatment. In healthy controls, no significant correlations were observed among clinical parameters, neuropsychological scores and metabolite ratios.

## Discussion

This study investigated the metabolic changes in the posterior parietal cortex and DLPFC brain regions in hyperthyroid patients before and after antithyroid treatment using ^1^H MRS. Our results showed significant changes in the major brain metabolites, mainly in the posterior parietal cortex region due to thyroid hormone dysfunction in hyperthyroid patients, which include (GPC + PCh) as a marker of cellular membrane turnover, Glu as a measure of glutamate metabolism, GSH as an oxidative stress marker and NAA as a neuronal cell marker (Fonnum, 1984).

It is known that thyroid hormones regulate the processes of terminal brain differentiation such as neurogenesis, neuronal migration, neuronal and glial cell differentiation, myelination, and synaptogenesis (Chan and Kilby, 2000; Bernal, 2007). Thyroid hormones mediate central nervous system effects primarily through interaction of the active hormone T3 with nuclear receptors and regulation of gene expression (Heuer, 2007; Bauer et al., 2008). In adult humans, thyroid hormone deficiency or excess may lead to brain metabolism changes that cause clinical manifestations, namely, neurological and psychiatric symptoms (Joffe and Sokolov, 1994; Bernal, 2007), which are usually reversible with proper treatment. Our findings have shown the metabolic changes in total choline, combined peak of Glu and Gln, and NAA or (NAA + NAAG) in the posterior parietal cortex and DLPFC in hyperthyroid patients.

In the current study, hyperthyroid patients had significantly lower (GPC + PCh)/tCr in both the posterior parietal cortex and DLPFC, compared to healthy controls, which reversed in both brain regions after antithyroid treatment. Choline acts as a precursor for major components of cellular membrane phospholipids such as phosphatidylcholine and sphingomyelin, and it affects cholinergic neurotransmission *via* the synthesis of the neurotransmitter acetylcholine (Bekdash, 2016). Choline also has pivotal functions, namely, the maintenance of structural integrity of membranes and modulation of cholinergic neurotransmission, functions that are often dysregulated in some neurodegenerative disorders (Bekdash, 2016). Thus, the reduced (GPC + PCh)/tCr might be due to reduced cholinergic neurotransmission as mediated by acetylcholine. Several studies have suggested that choline may attenuate age-related memory decline or memory impairments that may be induced during adulthood (Klein, 2000; Bekdash, 2016). We also found a positive correlation between (GPC + PCh)/tCr and memory and motor functions in the brain regions of hyperthyroid patients. Our study is in support of a previous study that has shown that the Cho/Cr ratio decreases when patients have thyrotoxicosis and increases after treatment when the conditions of patients change to euthyroidism (Bhatara et al., 1998). In addition, our study also showed a positive correlation of (GPC + PCh)/tCr in both the brain regions with certain memory and motor functions, and a negative correlation of (GPC + PCh)/tCr in DLPFC with fT3 in the hyperthyroid patients.

Our findings showed significantly reduced Glu/tCr and Glx/tCr ratios in the posterior parietal cortex in hyperthyroid patients compared to healthy controls. Glu is the principal excitatory neurotransmitter, whereas Gln is a precursor of Glu and GABA in the brain, and its metabolism depends on astrocytes (Fonnum, 1984; Erecinska and Silver, 1990). During neurotransmission, Glu is released from the presynaptic terminals and converted to Gln *via* the Gln synthetase, an astrocyte-specific enzyme, in astrocytes. The Gln travels back to the neuron, where it is reconverted to Glu by a phosphate-dependent glutaminase enzyme (Meldrum, 2000). Therefore, the decreased Glu/tCr, and Glx/tCr ratios as observed in our study might be due to abnormal Glu-Gln cycling or decreased Glu activity in hyperthyroid patients. After antithyroid treatment, a significantly decreased Glx/tCr ratio in both brain regions was found in the patients at follow-up. These results might indicate that the metabolite alterations persisted even after the patients returned to the euthyroid state following antithyroid treatment. A previous study has shown that reduced Glu and Gln in the parieto-occipital WM in patients with Graves’ thyrotoxicosis persistently decreased even after antithyroid treatment (Danielsen et al., 2008). Different responses in terms of metabolite changes were observed in the two cortical regions studied, wherein metabolites associated with Glu metabolism were altered only in the posterior parietal cortex region, depicting compartmentalization of thyroid hormone influence on astrocytic Glu metabolism in the brain. However, the disparity in Glu metabolism in different regions is still a question of research.

In addition, there was a significant increase in (NAA + NAAG)/tCr in DLPFC in the hyperthyroid patients as compared with controls. In the posterior parietal cortex, an increasing trend was observed in NAA/tCr in hyperthyroid patients. After antithyroid treatment, both NAA/tCr in the posterior parietal cortex and (NAA + NAAG)/tCr in DLPFC decreased but were not statistically significant. NAA is an essential amino acid that is released from the breakdown of NAAG and is mainly synthesized in the mitochondria. NAA takes part in many processes, like regulation of protein synthesis, lipid production, and the metabolism of aspartate and NAAG in the brain. Increased NAA/tCr and NAA + NAAG/tCr may suggest impaired catabolism of NAA, as NAA is catabolized in astrocytes and oligodendrocytes, and may be affected by astrogliosis (Baslow, 2003; Aston et al., 2005). Besides cellular alterations, studies have reported higher NAA indicating oxidative stress (Surendran and Bhatnagar, 2011; Khan et al., 2018). We also found a positive correlation between NAA/tCr and fT3 and fT4 in the posterior parietal cortex in patients at baseline and follow-up, and positive correlations between (NAA + NAAG)/tCr and fT3 and fT4 in DLPFC in patients at baseline only.

In our study, we found an increased GSH/tCr ratio, an important antioxidant of the cells in the hyperthyroid patients, which further showed an increasing trend in the patients at follow-up. It is reported that elevated thyroid hormones levels (hyperthyroidism) induce oxidative stress (Villanueva et al., 2013). An increase in GSH could possibly reflect either an upregulation of local GSH production or downregulation in GSH breakdown to counterbalance increased oxidative stress (Duffy et al., 2014). An MRS study has shown that patients with mild cognitive impairment (MCI) had elevated GSH levels in the anterior and posterior cingulate, and the higher levels of anterior cingulate GSH were associated with poorer cognitive performance in MCI patients (Duffy et al., 2014). This increase in GSH level was explained as an early compensatory or neuroprotective response in MCI (Duffy et al., 2014).

In this study, reduced mI/tCr was also observed in both the brain regions in hyperthyroid patients, but it was not statistically significant. Although a previous study has shown a significantly reduced mI/tCr ratio in the parieto-occipital WM and frontal GM in the acute phase of Graves’ thyrotoxicosis compared with healthy volunteers (Elberling et al., 2003). Another study reported that the mI/tCr ratio was increased in the parieto-occipital WM, occipital GM, and frontal GM in hyperthyroid patients after antithyroid treatment (Danielsen et al., 2008).

Our study has provided information on metabolic changes in hyperthyroidism. However, there are certain limitations to this study. Our sample size is small for the follow-up study. Future studies with a large number of subjects may improve the robustness of data. Secondly, a follow-up study at different time points is necessary to see if the complete reversal of brain metabolites after antithyroid treatment in hyperthyroid patients is possible. This information may be valuable in the management and therapeutic planning of hyperthyroid patients in the future. Thirdly, we have not assessed depression and anxiety scores, which may affect metabolite levels in hyperthyroid patients.

## Conclusion

Our study provides evidence that hyperthyroidism results in changes of key metabolites in the posterior parietal cortex and DLPFC, possibly indicating alterations in astrocyte physiology, glutamate/glutamine cycle, and/or oxidative stress in the adult human brain. In this study, altered neurometabolite ratios were found in the brain regions which showed reversible changes after antithyroid treatment in hyperthyroid patients. In addition, a few metabolites showed a significant correlation with thyroid indices (fT3 and fT4) and neuropsychological scores in the brain regions. The overall findings suggest that all the brain metabolites were not completely reversed in the hyperthyroid patients after antithyroid treatment, even after achieving euthyroidism. This finding has great relevance for the proper treatment and management of these patients.

## Data Availability Statement

The original contributions presented in the study are included in the article/supplementary material, further inquiries can be directed to the corresponding author/s.

## Ethics Statement

The studies involving human participants were reviewed and approved by INMAS-Institutional Human Ethics Committee. The patients/participants provided their written informed consent to participate in this study.

## Author Contributions

MK contributed to study design, protocol design, data acquisition, neuropsychological assessment, data processing, and approved the first draft manuscript. SS wrote and submitted the final manuscript. PR and SK contributed to manuscript review and study design. PK contributed to data acquisition. MD’S contributed to patient’s report preparation. RK contributed to recruitment and clinical assessment of thyroid patients. TS contributed to recruitment and clinical assessment of thyroid patients and study design. All authors contributed to the article and approved the submitted version.

## Conflict of Interest

The authors declare that the research was conducted in the absence of any commercial or financial relationships that could be construed as a potential conflict of interest.

## Publisher’s Note

All claims expressed in this article are solely those of the authors and do not necessarily represent those of their affiliated organizations, or those of the publisher, the editors and the reviewers. Any product that may be evaluated in this article, or claim that may be made by its manufacturer, is not guaranteed or endorsed by the publisher.
